# Supplementary material for: SomaVR: A low-cost virtual reality platform and implementation framework for medical education in resource-limited settings
Source: PLOS Digit Health. 2026 Feb 23;5(2):e0001253. doi: 10.1371/journal.pdig.0001253 (PMC12928446; doi:10.1371/journal.pdig.0001253)
Supplement: Attachment — Submitted filename: Response to Reviewers.pdf [file pdig.0001253.s001.pdf]

Dear Editor and Reviewers,

We thank you for the opportunity to submit a revised version of our manuscript for publication in PLOS Digital Health. We're grateful to you and the reviewers for the constructive feedback which has helped us to significantly improve the clarity and impact of our paper.

We have addressed all the points raised by the reviewers and have revised the manuscript accordingly. Below, we provide a point-by-point response to the reviewers' comments, detailing the changes we have made.

Reviewer #1:

This manuscript describes a set of mostly 360-videos that the team made to facilitate medical training. Although use of virtual reality to facilitate simulation-based learning for high-skill professions like medicine is promising, it is not clear what the goal of this manuscript is, how it differs from existing publications, and so little information about the included "studies" is provided that it is impossible to follow what was actually done or what the results are. Specific comments include:

We thank Reviewer #1 for their thorough review. We recognize that some parts of the initial submission did not sufficiently clarify the manuscript's primary goal, leading to a misunderstanding of its contribution relative to our previous publications. We have extensively revised the manuscript to make its purpose clear.

1.The introduction seems to suggest that this manuscript is solely focused on describing the VR system itself, but (as the authors point out) there are already several other papers published that detail the system and how it works. How is this manuscript different from these? What are its goals relative to these other papers?

This is a critical point, and we thank the reviewer for highlighting the need for this clarification. While we have previously published on the outcomes of two VR-based interventions (Buyego et al., 2022; Please et al., 2024), a key distinction is that those papers do not detail the technical system or how it works.

Our previous publications in journals such as BMC Medical Education and BMJ Open Quality were intentionally scoped for a clinical and educational audience, focusing squarely on the feasibility, educational outcomes, and qualitative user experiences of the interventions. To include deep technical specifications in those manuscripts would have been out of scope, making them unwieldy and detracting from their primary message.

The motivation for the present manuscript arose directly from the success of those studies; we received numerous inquiries from other institutions in LMICs asking for a

practical guide on how they could replicate our work. This manuscript is our answer to those requests. It is a dedicated technical piece, written specifically for a digital health audience like that of PLOS Digital Health, that provides the first comprehensive description of the SomaVR platform's technical architecture, the actual software, the software development process, cost-benefit analysis, scalability considerations, and implementation strategies. It is intended to serve as a replicable blueprint for others. We have substantially revised the Introduction section to make this contribution and purpose much clearer. (Page 5, lines 20 – 27)

2.Basic, essential information about the two "studies" that the authors refer to is missing, such that the rigor of this work cannot be evaluated. For example, the number of participants in each study, eligibility criteria, and basic demographics are not reported. There is no information about what outcomes were assessed in either study or how, or whether the methods used to assess those outcomes were valid. There is no information about whether participants were randomized, and the two studies appear to use different methods without explanation. The ethics subsection says consent was obtained from all participants but then says "As this study involved the secondary analysis of this already published and anonymized data, no further ethics approval or consent was necessary." Which is it?

We thank the reviewer for this important feedback. We agree that providing key context for the validation studies is essential. To address this, we have taken a two-part approach. First, we feature Figure 3, which provides a clear, side-by-side comparison of the two case studies across eight key domains (e.g., participants, hardware, setting). Second, to supplement this visual summary with the specific methodological details the reviewer requested, we have added new text to Section 2.8. This new text now explicitly states the study design for each case (e.g., randomized design for Study 1, observational for Study 2) and clarifies the primary outcomes that were assessed, with clear citations to the original papers for exhaustive details. We believe this combined approach provides the necessary context for the reader, while maintaining the manuscript's focus on the technical framework. (Page 11, lines 33 – 35)

Regarding the ethics statement, we have revised it for clarity. The statement now correctly explains that this manuscript analyzes previously published, anonymized data (requiring no additional consent), while the original studies themselves obtained all appropriate institutional approvals and participant consents. (Page 13, lines 3 – 7)

3. Similarly, the results are impossible to follow. Of the two results presented, it is not clear what either is actually evaluating. For example, Fig. 6 purports to show "VR training scores," but training on what? What is this assessing? The other result focusing on the "ease of use" of the VR seems of limited import. No other results discussed in the Methods section (e.g., feasibility, knowledge retention, satisfaction, etc) are reported.

We thank the reviewer for this feedback. The results are now presented in a logical order that aligns with the paper's objectives. We first describe the technical output of

our work. We then present key outcomes from the case studies (e.g., training scores and user-reported ease of access) as direct evidence of the platform's effectiveness and usability.

As also suggested by Reviewer #2, we have also corrected the figure order mismatch and have rewritten the figure captions to be more descriptive, clarifying exactly what each figure shows and how it was derived. (Page 16 and 17)

4.Although most of the manuscript makes it sound like one system or one consistent set of experiences, the use of very different hardware systems with different properties to expose users to different experiences for different applications makes it very difficult if not impossible to understand what is actually being evaluated in the study, and so, what the results mean. Google Cardboard is very different than HTC Vive systems, which are in turn very different than Oculus Quests.

We appreciate the opportunity to clarify this point. The use of diverse hardware is not a methodological flaw but a core feature and strength of our platform implementation framework. We have revised the Methods and Discussion sections to emphasize that the framework was deliberately designed to be adaptable to a wide range of hardware from high-end systems like the HTC Vive to low-cost solutions like Google Cardboard with a smartphone. This flexibility is crucial for successful deployment in LMICs, where available technology varies greatly.

Furthermore, in Section 2.2, we now explain that different pedagogical goals require different types of VR experiences. Some concepts are best delivered via passive 360-degree video (which can run on simple hardware like a smartphone), while others require fully interactive simulations (which demand more powerful headsets). Our framework is designed to accommodate this reality, allowing educators to select the appropriate technology for the specific learning objective. The case studies exemplify this, demonstrating how different content types were deployed on different hardware to meet specific training needs. (Page 6, section 2.2)

5.It is not clear whether any of the content involved in the system was truly interactive, as the only parts described in detail focus on 360 video. Relying entirely on 360 video has important limitations as a training tool because it does not enable interaction. Interaction is essential for capturing the benefits of simulation-based learning.

The system is interactive. We have added a description of the interactive elements in sufficient detail. We have expanded Section 2.2 to include concrete examples and Section 2.7 (SomaVR system architecture). Specifically, we now describe how the COVID-19 IPC module included a fully interactive simulation where learners were required to perform procedures (like donning and doffing PPE). The system provided real-time feedback via audio cues for incorrect actions and would not allow the user to advance until the step was performed correctly. We also provide more detail on the interactive, multiple-choice assessments that were embedded directly into the

360-degree video streams. This provides clear evidence of interactivity that goes far beyond passive viewing. (Page 6, section 2.2)

6.The manuscript frequently uses excessively venerating phrases like "seamless viewing experience" and that "experiences were meticulously crafted," without explaining how, which makes the manuscript read like an advertisement for the system rather than an evaluation.

We have performed a thorough review of the manuscript and have revised and removed overly subjective phrases.

Reviewer #2: plos Digital health D-25-00093
SomaVR: low cost VR platform

General:
This is a well-scoped, well-written report on valuable research programs. The findings have substantial relevance to health care training in LMICs. They are well-suited to publication in PLOS Digital Health.

Example of relevance: Our lab has explored AR as a means to test-drive deployments of new gear to remote locations, and the findings in this paper are valuable to our efforts.

The paper, in my opinion, requires very little work to be ready for publication. Below I list a few odds and ends. The comments about section 3.3 are most important.

Comment for PLOS Digital Health: Line numbers make review much easier. Is there a way to require these in the template for submissions?

We are very grateful to Reviewer #2 for their positive and encouraging feedback and their valuable suggestions for improving the manuscript. We have incorporated all of their recommendations.

Comments for authors:

1. "as follow-up to Financial Disclosure": fill out if required (currently empty)

Thank you for noting this. We will ensure the financial disclosure section is completed in the final submission to the journal.

2. "recent review by Mergen et al" add reference numbers

We have added the reference number [12] for the Mergen et al. review in the Introduction.(Page 4, line 32)

3. "SomaVR's potential to improve ...": also: long-distance knowledge exchange

This is a great point. We have added "long-distance knowledge exchange" to the Introduction when discussing the potential of the SomaVR platform, as this was a key feature of our surgical training study. (Page 5, line 16)

4. Section 2.2: Perhaps you could add brief definitions of VR and AR, since this is not clear to all readers.

We agree this is a helpful suggestion. We have added a brief definition of Virtual Reality (VR) at the beginning of Section 2.2. To maintain the manuscript's focus on the technologies directly used in our work, we have opted not to include a definition for Augmented Reality (AR), as it falls outside the scope of this paper. (Page 6, lines 21 – 23)

5. 2.3, 2.6: Great mix of expertise on team

We thank you for this comment!

6. 2.7.1 (1): missing "and"

We thank you for this comment. This has been added.

7. 2.7.1: Perhaps you could include examples of videos in an online S.I. (if you have not already)

This is a wonderful suggestion. We will prepare supplementary video files to be included with the final submission, showcasing examples. We have also added a video to the GitHub link

8. 2.7.1: Please include github link here as well as (or instead of) in "Data and Code Availability".

We have now included the GitHub link directly in Section 2.7.1 for easier access for the reader, in addition to the Data and Code Availability section.(Page 10, lines 1 – 3)

9. Study 2: Please add a sentence to clarify that there was no test cohort vs control cohort structure to this study, but that it was observational. (relevant since study 1 did have test-control cohort structure).

Thank you for this suggestion. We have added a sentence in the description of Study 2 in Section 2.8 to clarify that it was an observational study assessing user experience and skill acquisition, unlike Study 1, which used a comparative cohort structure. (Page 11, lines 33 – 35)

10. 3.1: "allows repeated practice in a safe...": I imagine this would be valuable to have before tangling with a Marburg outbreak.

Absolutely! Our Infectious Diseases training department has found these training very useful.

11. 3.3: A few things:

Fig 6 and 7 appear to be switched. I suggest giving results in the same order as the experiments are described in Section 2.8. This is clearer for the reader.

We sincerely thank the reviewer for identifying these errors. We have implemented all of these suggestions:

- The order of the figures has been switched
- The captions have been revised

Current Fig 6: It would help the reader if you clarified that panel (a) is a subset of panel (b) (perhaps switch the 2 panels for a more natural left-to-right reading).

Thanks for this brilliant comment! We've adjusted the figure now as advised, starting from left to right and also clarified in the caption that it's a subset of panel a

Current fig 6 caption: "the untrained cohort" is confusing, since they did receive traditional classroom training. Perhaps refer to this cohort as "control cohort".

Thank you! The figure caption has been modified and now read as "control cohort"

Good limitations section

Good conclusion: "Our approach etc"

Thank you for an interesting paper!

Thank you very much for your kind words!
