# Supplementary material for: SomaVR: A low-cost virtual reality platform and implementation framework for medical education in resource-limited settings
Source: PLOS Digit Health. 2026 Feb 23;5(2):e0001253. doi: 10.1371/journal.pdig.0001253 (PMC12928446; doi:10.1371/journal.pdig.0001253)
Supplement: Attachment — Submitted filename: Response_to_Reviewers_auresp_2.pdf [file pdig.0001253.s002.pdf]

Dear Editor and Reviewers,

We thank you for the opportunity to submit a revised version of our manuscript for publication in PLOS Digital Health. We're grateful to you and the reviewers for the constructive feedback which has helped us to significantly improve the clarity and impact of our paper.

We have addressed all the points raised by the reviewers and have revised the manuscript accordingly. Below, we provide a point-by-point response to the reviewers' comments, detailing the changes we have made.

Reviewer #2
All good!

Thank you!

Reviewer #3
This manuscript presents SomaVR, an affordable and modular virtual reality (VR) training platform, developed specifically for medical education in low- and middle-income countries (LMICs). The work is timely and relevant, responding to persistent gaps in training infrastructure and workforce development across resource-limited settings. The integration of 360-degree video and interactive VR modules, combined with open-source code availability and capacity-building strategies, represents a compelling and replicable approach.

The authors are commended for presenting not only the technical development but also two applied case studies (COVID-19 infection prevention and surgical training). These add practical validation and highlight the potential impact of the SomaVR framework. The manuscript is well-written, logically organized, and clearly grounded in the global health context.

We thank Reviewer #3 for their thorough and encouraging review. We appreciate the recognition of SomaVR's originality and the constructive suggestions for stregthning the manuscript. Below we address each comment.

However, there are areas requiring minor revision to improve methodological clarity, strengthen the evaluation component, and streamline the presentation. Below are detailed comments to guide your revision:

1. Significance and Originality
The concept of a low-cost, scalable VR framework for LMICs is original and well justified.

The approach stands out by combining hardware flexibility, offline delivery capability, and a sustainable local developer model.

Suggestion: Consider briefly comparing SomaVR to other LMIC-focused VR platforms, if any exist, to better position its novelty.

Thank you for this comment, we've added a paragraph in the Introduction section stating previous VR projects that have been done and these challenges that have been encountered and how our framework/tool addresses some of them (Page 5, lines 2 – 10)

2. Methodological Rigor
The description of software architecture and hardware setup is very detailed and helpful.

However, the evaluation design lacks methodological transparency. Please provide:

Clarification on how participants were selected and grouped (randomized or not).

Descriptions of assessment tools used in pre-/post-testing.

Statistical analysis details: were significance tests performed? Were effect sizes calculated?

Actionable Recommendation:
Add a table summarizing key features of the two evaluation studies (sample size, duration, modality, metrics, statistical tests, key results).

We appreciate this feedback. We have added a brief summary of the methodological descriptions in Figure 3 and also added clarifications in Section 2.8(Implementation and Evaluation Studies) about

study design, sample size, device type, participant type among others. For readers who may need extra details about the individual studies, we've added a statement directing them to our 2 primary published studies as well. (Page 13, 1 – 10)

3. Results and Data Presentation
The training outcomes are presented clearly, and the figures illustrate core findings effectively.

However, Figures 6 and 7 need better labeling and contextual explanation:

Define "trained" and "untrained" cohorts directly in figure captions.

Indicate sample sizes, statistical significance (if any), and data collection methods.

Recommendation: Consider adding a brief comparative summary of knowledge acquisition scores or engagement indicators across the two cohorts.

Thanks for this comment, the figures show a comparison of the performance scores across the 2 cohorts. We've also added a clarification in Figure 7 legend stating the sample size (Page 19)

4. Technical Description of SomaVR
The software architecture section is detailed and informative. The use of Unity3D is appropriate, and the breakdown of its components is helpful.

The Enduvo integration is a strength, as it supports blended didactic and procedural content.

Minor Suggestion: Some subsections (2.7.1) could be more concise to avoid redundancy. For example, interaction mechanisms and platform support are discussed in multiple places with overlapping phrasing.

We appreciate this observation. We have reviewed Section 2.7.1 and condensed redundant descriptions while maintaining technical clarity (Page 10, section 2.7.1)

5. Discussion and Implications
The Discussion section is thoughtful, especially regarding costs, accessibility, and motion sickness mitigation.

The authors rightly emphasize the challenge of VR developer availability in LMICs and their solution via capacity-building is commendable.

Enhancement Suggestions:

Discuss possible limitations in learner evaluation (e.g., bias, lack of long-term follow-up).

In the Future Work section, consider listing more concrete next steps (e.g., number of modules to be created, training hubs planned).

We appreciate these suggestions and have enhanced our Future work sections with next steps that include addition of extra modules in different medical specialties (Page 22).

6. Clarity and Structure
Overall writing quality is strong. The manuscript is well-organized and flows logically.

That said, the Methods and Discussion sections are overly long. Some compression would improve readability.

Language edits:

Replace redundant phrasing (“immersive and interactive” is used excessively).

Ensure consistent terminology for technologies (e.g., use “360-degree video” consistently).

We appreciate this feedback and we’ve overall improved clarity in the manuscript.

7. Ethics, Open Science and Data Sharing
Ethical considerations are appropriately addressed and conform to best practices.

Open sharing of the SomaVR code on GitHub is commendable and aligns with FAIR principles.

Improvement Suggestion:
Consider archiving the GitHub repository using Zenodo or a similar service to obtain a permanent DOI.

We appreciate this suggestion. The code for the platform is published on GitHub (https://github.com/aceuganda/somavr) and the detailed documentation on how to run it is also provided on the GitHub page. The GitHub link is provided in the manuscript

8. Figures, Tables, and Supplementary Materials
Figures 1–5 are informative. Figure 1 (SomaVR framework) could benefit from clearer labeling of each module.

Table 1 (Cost breakdown) is relevant. Adding a “Reuse/Usability” or “Learning Curve” column would increase practical value.

Figures 6 and 7 are important but currently not fully self-explanatory. Improve captions and add statistical context if available.

We thank you for the feedback, we’ve added clarifications in our figure captions for Fig 6 and 7 (Page 18 – 19).

Conclusion
This manuscript offers a valuable contribution to global digital health and medical education. With minor revisions focused on methodological transparency, concise writing, and clarity in results presentation, this work will be of high interest to the readership of PLOS Digital Health.

Reviewer #4
Summary
This paper presents a framework for implementing low-cost VR-based education infrastructure in low- and middle-income countries (LMICs), with case studies focused on COVID-19 infection prevention and surgical training. The study offers valuable insights into the feasibility of using existing VR technologies in resource-constrained settings.
As a computer scientist, I approached the paper with particular interest in the technical and organizational aspects. From this perspective, I find the overall concept and contribution important and timely. However, the technical components, including system setup, infrastructure requirements, and cost breakdowns, require further clarification and refinement to fully support replication and implementation.

We thank Reviewer #4 for their thoughtful and constructive feedback. Their technical perspective has been invaluable in strengthening the practical implementation guidance in our manuscript. Below we address each comment:

Major Comments
1.Clarity and Structure of Methods Section
The methodology section would benefit from reorganization. I recommend presenting the content from abstract to concrete: start with administrative and organizational aspects, then progress to infrastructure, hardware, systems, and finally evaluation. This structure would improve clarity and flow, especially for readers seeking practical implementation guidance.

We agree that this reorganization will improve clarity and flow, particularly for readers seeking practical implementation guidance. We have restructured the Methods section with the following new order:

2.1 Team composition (moved from 2.3)
2.2 Content development and curriculum design (moved from 2.4)
2.3 Collaborations (moved from 2.6)
2.4 Internet requirements (moved from 2.5)
2.5 Hardware devices used (revised from 2.1)
2.6 Defining Virtual Reality categories (moved from 2.2)
2.7 SomaVR system architecture (unchanged position)
2.8 Implementation and Evaluation Studies (moved from 2.8)

This restructure now flows from organizational aspects (team, curriculum, partnerships) to infrastructure (internet, hardware) to technical systems (software architecture) to evaluation (Page 5 to page 9).

2.Inconsistent Terminology on Content Delivery
There is a lack of clarity regarding whether the training content was delivered live or asynchronously. Section 2.1 mentions "live content," while Section 2.4 describes the surgical training as using "entirely online content." If the content was live, was it also recorded? If not, why was asynchronous access not provided?

We appreciate this observation and recognize the terminology may have been confusing. The content was indeed delivered live (real-time streaming) but accessed online (via internet connection). To clarify this distinction, we have made the following changes (now section 2.2 and added clarifying text:

"…For the surgical training study, content was delivered as live-streamed demonstrations, enabling real time viewing by participants in Kampala and remotely. This live delivery approach was chosen to facilitate immediate knowledge transfer from UK-based surgical instructors to Ugandan learners. Recordings were subsequently made available for review, allowing participants to revisit procedures… "

The use of 'entirely online content' in our original text referred to the streaming delivery method (requiring internet connectivity) as opposed to the COVID-19 IPC study's pre-downloaded offline content.

In Section 2.4 (Internet requirements), we have clarified:
"In contrast, the surgical training study relied ***on*** *live-streaming content, necessitating a stable and high-bandwidth internet connection during the training sessions."*

3.Missing Technical Specifications
For a technically oriented audience, the paper lacks essential details on system requirements. Specifically:
oWhat are the average file sizes for downloadable content in Study 1?
oWhat is the minimum bandwidth needed to support live streaming in Study 2?
These specifications are necessary for readers aiming to replicate the project in LMIC settings.

Thank you for this point. We acknowledge that some minimum requirements were not explicitly stated. We have added a new subsection 2.4.1 called Technical Specifications which now lists these specifications (Page 8, lines 21 - 34)

4.Team Size and Resources
Please provide more information on the deployment teams:
oHow many people were involved?
oWhat was the estimated time commitment (e.g., person-hours) per team?
This would help assess the scalability and resource implications of the proposed framework.

We agree this information is essential for assessing scalability. We have expanded Section 2.1 to include team size and time commitments(Page 6, Section 2.1)

5.Cost Reporting
While the breakdown of VR-related costs is helpful, the paper does not include estimates for internet access or personnel costs. These are significant components in LMIC implementations and should be addressed, at least at a high level.
We appreciate this observation regarding comprehensive cost reporting. We agree that personnel and internet costs are important implementation considerations. However, quantifying these precisely in our context is challenging due to following reasons:

- Internet access: Our studies were conducted within a university setting with support from the Research and Education Network Uganda (RENU), the university's official service provider. Internet access were provided institutionally rather than billed per project. Consequently, no separate cost item was attributable. Given that internet prices vary widely across LMICs, we have chosen to report bandwidth specifications (10–25 Mbps per user; added in Section 2.4.1) so that institutions can use these figures to obtain local estimates.

- Personnel costs: The VR development and implementation teams comprised institutional staff at ACE-Makerere whose roles spanned multiple projects, making it impractical to isolate exact salary allocations. To enhance transferability, we have now added person-hour estimates following your comment #4 (Added to Section 2.1 in detail: 800–1000 hours for the IPC study; 300–400 hours for the surgical training), we hope this will offer a measure for resource planning.

To also make it clearer, we have updated Table 1's caption with a note summarizing this point and stated that only hardware costs are presented and a reference to the internet and personnel requirements.

Minor Comments

•Chapter 2.1 Scope and Focus
The current title („Hardware") is overly broad and does not clearly reflect the structure of the paper. Consider emphasizing the organizational implementation first, and relocating the hardware-focused discussion to a later section.

Thank you for this comment. We renamed the section to "Hardware devices used" Formerly section 2.1, we have now moved it to 2.5 and now appears after organizational and infrastructure sections.

•Figure 1 Legend
The meaning of the colors used in Figure 1 is not explained. Please include a legend or clarify this in the figure caption.
Thank you for this comment. We have enhanced the figure caption to explain the color coding.

•Use of Standardized Evaluation Tools
For future work, I recommend employing established tools to evaluate user experience and technology adoption, such as:
oUser Experience Questionnaire (UEQ-S)
oUnified Theory of Acceptance and Use of Technology (UTAUT)
Their use would strengthen the evaluation component and allow for cross-study comparisons.

Thank you for this excellent suggestion. We will consider these for our future studies and we've added a note in our Future Work section.

Conclusion

The paper addresses an important topic with strong relevance to global health and education. However, in its current form, the technical and methodological sections require significant improvement for the work to be replicable and broadly impactful. I encourage the authors to revise with attention to the comments above, especially those related to clarity, technical detail, and completeness of cost and deployment data.
